# Spatial Proteomics as a Potential Decision‐Support Layer for Early Melanoma: A Narrative Review

**DOI:** 10.1111/ijd.70665

**Published:** 2026-09-29

**Authors:** Leticia Szadai, Jeovanis Gil, György Marko‐Varga

**Affiliations:** ^1^ Department of Dermatology and Allergology, Albert Szent‐Györgyi Faculty of Medicine University of Szeged Szeged Hungary; ^2^ Department of Translational Medicine Lund University Lund Sweden; ^3^ Department of Biotechnology College of Life Science and Biotechnology, Yonsei University Seoul Korea; ^4^ 1st Department of Surgery Tokyo Medical University Tokyo Japan

**Keywords:** borderline melanocytic lesions, early stage melanoma, patient risk stratification, single cell technologies, spatial proteomics, spatial transcriptomics

## Abstract

Distinguishing early melanoma from borderline, atypical, or biologically intermediate melanocytic lesions remains one of the most consequential and urgent diagnostic challenges in dermato‐oncology, since melanoma causes nearly 95% of skin cancer deaths. This narrative review establishes the biological relevance and evaluates the clinical maturity of mass spectrometry‐based proteomics, with particular emphasis on spatially resolved approaches, as a powerful strategy to address this critical diagnostic gap. We position spatial proteomics within the broader landscape of DNA‐, RNA‐, and protein‐based molecular diagnostics for functional manifestation, highlighting that genomic and transcriptomic methods offer standardized, higher‐throughput workflows but limited insight into the tumor microenvironment and functional protein‐level biology. Laser capture microdissection–mass spectrometry, imaging mass spectrometry, and Deep Visual Proteomics each preserve spatial and single‐cell resolution. However, protein expression is inherently plastic, shaped by microenvironment, tissue handling, and technical artifact, and no existing study has been powered specifically for the diagnostically indeterminate categories where clinical need is greatest. We discuss how compartment‐resolved “mitochondrial‐high” profiles and “immune‐low” microenvironment states, evidence largely extrapolated from progression and metastatic biology, might inform future risk stratification. Spatial proteomics represents a biologically promising, but not yet clinically validated, extension of morphology‐led melanoma diagnosis, with the potential to resolve functional states within annotated tumor compartments. Integrated with AI‐guided tissue annotation, compartment‐resolved mass spectrometry and spatial proteomic profiling could, following prospective validation, help improve the distinction between borderline melanocytic lesions and melanoma while refining patient risk stratification.

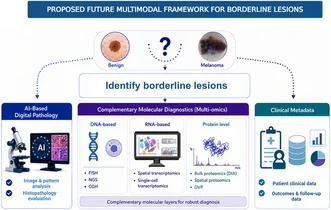

## Graphical abstract

This schematic illustrates a proposed, future multimodal diagnostic framework in which spatial proteomics, AI‐based digital pathology, and clinical data would be integrated to support the evaluation of borderline melanocytic lesions. The figure depicts a conceptual research direction rather than a workflow currently available or validated for clinical decision‐making.

**Table:** 

Key Questions	Author Insights
Does the dynamic, microenvironment‐dependent nature of proteomic expression preclude a stable diagnostic signature for early melanoma—or does that plasticity offer diagnostic information that static genomic markers cannot?	Rather than representing only a source of variability, proteomic plasticity may provide complementary functional information by capturing active tumor states and tumor‐microenvironment interactions that static genomic or transcriptomic markers cannot. Spatial proteomics therefore has the potential to add a functional layer to morphology‐based diagnosis, provided its findings are interpreted within the appropriate clinical context.
If proteomic profiles exist on a continuum rather than in discrete benign and malignant clusters, how should clinicians translate probabilistic findings into binary treatment decisions?	Spatial proteomics should not function as a stand‐alone diagnostic test. Instead, continuous proteomic risk profiles will require prospectively validated thresholds and should be integrated with histopathology, immunohistochemistry, molecular diagnostics, AI‐assisted digital pathology, and clinical context to support, rather than replace, diagnostic and therapeutic decision‐making.

## 1 Introduction

Early melanoma represents a significant diagnostic challenge, as the key clinical issue is not the identification of obvious melanoma, but its distinction from borderline, atypical, or biologically intermediate melanocytic lesions. Although melanoma accounts for only approximately 1%–2% of all skin cancers, it is responsible for the vast majority of skin cancer–related deaths, highlighting the importance of timely and accurate diagnosis [1]. Beyond its clinical consequences, melanoma also imposes a substantial economic burden, largely driven by the high cost of immune checkpoint inhibitors used in the treatment of advanced disease [2]. Consequently, improving the accurate distinction between early melanoma and benign or borderline melanocytic lesions has important implications not only for patient outcomes but also for optimizing healthcare resource utilization and reducing unnecessary treatment.

Yet the biological behavior of an individual lesion remains genuinely difficult to predict at the time of diagnosis. Most lesions remain indolent, while a minority progress rapidly, and current diagnostic tools cannot reliably distinguish between these entities at the time of biopsy. In clinical practice, diagnostically challenging melanocytic lesions are evaluated by histopathological examination using hematoxylin and eosin staining, supplemented by immunohistochemistry when indicated. Although early diagnosis remains the cornerstone of melanoma management, the continuous rise in melanoma incidence has not been accompanied by a proportional reduction in mortality [3, 4]. This discrepancy has raised concerns regarding overdiagnosis and highlights a critical unmet need, not simply to detect more melanocytic lesions, but to distinguish biologically aggressive melanomas from indolent or borderline lesions that may never progress. This raises an important question: What characteristics should an ideal complementary decision‐support tool possess for the evaluation of borderline melanocytic lesions and the early detection of melanoma?

In the era of digital twins [5] and artificial intelligence (AI)‐driven multimodal patient stratification [6], advances in precision oncology have accelerated the development of spatial and single‐cell technologies that complement conventional histopathology. Among these, mass spectrometry (MS)‐based proteomics, particularly spatially resolved proteomics, provides functional molecular information beyond morphology and has emerged as a promising tool for improving the diagnosis of early and borderline melanocytic lesions. Its clinical potential has been substantially enhanced by the convergence of large, clinically annotated biobanks, artificial intelligence‐assisted digital pathology, advanced molecular diagnostics, and computational analyses, which together establish a multimodal precision oncology framework for integrating proteomic, molecular, and morphological data to improve biomarker discovery, validation, and the diagnosis of early and borderline melanocytic lesions [5].

This review distinguishes biological discovery from clinical applicability, asking not only whether proteomics is biologically relevant to melanoma but also whether it is sufficiently mature to support the routine diagnosis of early and borderline melanocytic lesions. We examine how mass spectrometry‐based proteomics can be integrated with histopathology, immunohistochemistry, molecular diagnostics, and AI‐assisted digital pathology within a multimodal diagnostic framework, while highlighting its potential to improve patient stratification, refine risk assessment, and ultimately reduce diagnostic uncertainty in precision oncology.

## 2 Emerging Diagnostic Technologies and Their Relevance in Early Melanoma Studies

From a clinical perspective, the management of a suspicious melanocytic lesion begins with a fundamental decision following clinical and dermoscopic evaluation: Whether the lesion can be safely monitored or should be surgically excised. Since melanoma exhibits morphological and biological heterogeneity, a suspicious melanocytic lesion is generally excised for histopathological evaluation, which is the gold standard for melanoma diagnosis [2]. When histopathological examination confirms a benign diagnosis, no further therapeutic intervention is required, and routine clinical surveillance is generally sufficient. Whereas, if melanoma is confirmed, it initiates a well‐established diagnostic and therapeutic pathway that may include molecular testing, such as assessment of mutation status by PCR or NSG (next‐generation sequencing), multidisciplinary tumor board discussion, and stage‐specific treatment planning in accordance with current clinical guidelines [2].

The greatest diagnostic challenge arises when histopathological findings remain equivocal despite expert evaluation. Although immunohistochemistry (e.g., PRAME [7], Ki‐67 [8]) can help characterize the borderline lesions, they do not always provide a definitive diagnosis due to morphologic limitations and may increase diagnostic turnaround time [8]. This diagnostic gap has driven the emergence of spatial and single‐cell technologies that complement conventional pathology by providing high‐resolution molecular and functional characterization of melanocytic lesions beyond morphology alone (Figure 1).

**FIGURE 1:**
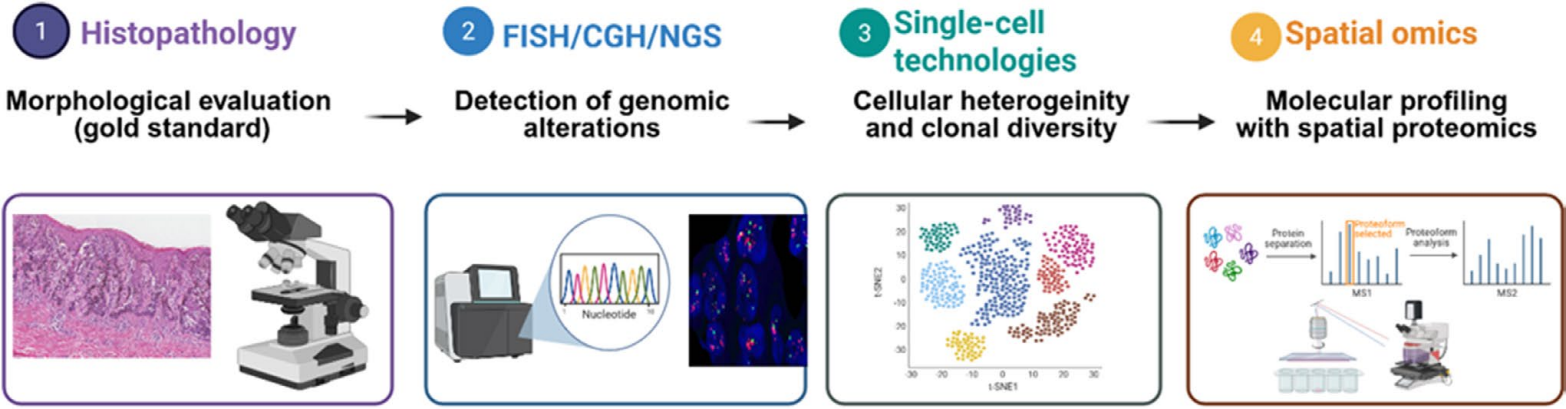
From morphology to multi‐omics integration. Each technological advance builds upon the previous one, adding complementary layers of information for a deeper understanding of melanoma biology. The figure was created with BioRender [9].

### 2.1 DNA‐Based Diagnostic Approaches

To understand where spatial and single‐cell technologies, especially spatial proteomics, fit within the broader landscape of emerging molecular techniques, it is useful to consider these approaches that are increasingly applied in early melanoma research: DNA‐, RNA‐, and proteome‐based molecular diagnostics. These technologies differ in three key characteristics relevant to diagnostic applications: The measurement of molecules, sample throughput, and the preservation of spatial tissue information.

Genome‐based approaches have substantially advanced the molecular diagnosis of melanoma and currently represent the most established complementary molecular tools in dermatopathology [10]. For example, polymerase chain reaction (PCR) remains the gold standard for detecting BRAF mutations [11], while next‐generation sequencing (NGS) enables the simultaneous assessment of multiple melanoma‐associated mutations beyond single‐target PCR analyses [12]. In addition, comparative genomic hybridization (CGH), which enables genome‐wide detection of chromosomal copy number variations, and fluorescence in situ hybridization (FISH), which identifies copy number alterations at predefined genomic loci, have demonstrated clinical value in distinguishing melanocytic lesions across the spectrum from benign nevi to metastatic melanoma, thereby supporting the evaluation of diagnostically challenging lesions [13]. These methodologies offer standardized workflows, relatively higher throughput, and greater accessibility for routine diagnostic implementation [12, 14]. However, they primarily detect genomic alterations and provide limited insight into the tumor microenvironment, intratumoral heterogeneity, and the functional activation of downstream signaling pathways (e.g., post‐transcriptional regulation, post‐translational modifications, and environmental influences) [15] (Table 1).

**TABLE 1:** Emerging molecular approaches for distinguishing borderline melanocytic lesions.

Approach	Clinical application	Molecule measured	Sample	Approx. cost per sample*	Aim	Limit
				Approx. turnaround time*		
FISH/CGH/NGS	Routine/Complementary	DNA	FFPE, FFT	Low to moderate, Days to weeks	Detection of driver mutations, chromosomal copy number alterations	Limited functional information
Spatial transcriptomic	In research	RNA	FFPE (more platforms, e.g., Visium), fresh tissue	High, Weeks	Spatial gene expression architecture	Measures mRNA rather than proteins; limited functional information and high cost.
Single‐cell RNA sequencing	In research	RNA	Fresh tissue	Moderate to high, Weeks	Cellular transcriptomic heterogeneity	Imperfect proxy for protein abundance/activity, requiring higher costs and advanced computational tools
Bulk proteomics	Emerging	Protein	FFPE, fresh tissue	Low to moderate, Days to weeks	Deep proteome coverage	Dilutes localized signals
Spatial proteomics	Translational	Protein	FFPE, fresh tissue	Moderate to high, Days to weeks	Links molecular signal to histological structure	Lower depth than bulk MS at high spatial resolution; technically demanding
Single‐cell proteomics	In research	Protein	Fresh tissue (freshly isolated cells)	High, Weeks	Cellular functional heterogeneity	Very low protein input per cell limits coverage; spatial context usually lost unless imaging‐guided

Abbreviations: approx.—approximately; FFPE—formalin‐fixed, paraffin‐embedded; FFT—fresh frozen tissue, MS—mass‐spectrometry.

* Estimated costs and turnaround time are approximate and may vary depending on the analytical platform, laboratory infrastructure, and the number of samples processed simultaneously batch size.

### 2.2 RNA‐Based Diagnostic Approaches

To complement DNA‐based diagnostics, RNA‐based molecular assays have emerged to capture the transcriptional consequences of genomic alterations and provide additional biological information relevant to early melanoma detection. Recently, the Pigmented Lesion Assay (PLA), a noninvasive adhesive‐patch test performed on intact, unbiopsied lesions that assesses LINC00518 and PRAME expression; the myPath Melanoma 23‐gene expression assay; and NGS‐based molecular classifiers based on biopsy tissues have shown promising diagnostic accuracy for borderline melanocytic lesions [16]. Nevertheless, these assays remain largely confined to specialized centers because additional prospective validation, standardization, and demonstration of clinical utility are required before routine implementation in clinical practice [16]. Moreover, they evaluate gene expression at the bulk tissue level, thereby averaging signals across heterogeneous cell populations and providing limited information on cellular heterogeneity and the spatial organization of the tumor microenvironment. To overcome these limitations, single‐cell RNA sequencing (scRNA‐seq) was developed to resolve gene expression at the individual cell level to characterize different cell types, states, and subpopulations [17, 18]. In melanoma, single‐cell RNA sequencing has been shown to distinguish malignant from non‐malignant cell populations while simultaneously resolving distinct cellular states within the tumor microenvironment, thereby providing insight into intratumoral heterogeneity [19]. An important advantage of single‐cell RNA sequencing is its high cellular throughput, enabling the simultaneous profiling of thousands to tens of thousands of individual cells per sample [20] (Table 1), which substantially exceeds the throughput currently achievable with single‐cell proteomics. Although the cost per profiled cell has decreased considerably, the overall cost per sample remains relatively high owing to extensive library preparation, sequencing, and bioinformatic analyses [20]. Moreover, this approach typically requires tissue dissociation, resulting in the loss of the spatial relationships between cells that are essential for understanding tumor architecture and microenvironmental interactions [17].

Spatial transcriptomics addresses the loss of spatial information by mapping transcript expression directly onto tissue sections by capturing a number of cells per spot. Widely used spatial transcriptomic platforms can be broadly grouped into three categories: sequencing‐based approaches (e.g., 10× Genomics Visium), which offer whole‐transcriptome coverage at spatial resolution; probe‐based methods (e.g., NanoString, GeoMx), which use barcoded oligonucleotide‐conjugated probes to quantify predefined transcripts within manually selected regions of interest; and imaging‐based approaches (e.g., CosMx, MERFISH), which employ cyclic fluorescence in situ hybridization to visualize targeted transcripts at near‐single‐cell or subcellular resolution [21]. By preserving tissue architecture while profiling spatial gene expression, spatial transcriptomics enables the identification of spatially resolved transcriptional signatures associated with melanoma progression and improves the molecular characterization of diagnostically challenging melanocytic lesions [22, 23, 24] (Table 1). Despite its high cellular throughput [21], major barriers to the widespread clinical implementation of spatial transcriptomic technologies remain, including high per‐sample costs [25], relatively long turnaround times (several weeks or longer for complex workflows), and substantial computational requirements [26] (Table 1). More fundamentally, transcript abundance does not necessarily reflect protein abundance or cellular function, as gene expression is further shaped by post‐transcriptional regulation, post‐translational modifications, and environmental influences acting both within and outside the cell [27]. Consistent with this, large‐scale proteogenomic studies have demonstrated only a moderate correlation between mRNA expression and protein abundance in tumors [28], highlighting the limited concordance between transcriptomic and proteomic data. These findings underscore the added value of proteomics, which captures the dynamic functional state of the tumor beyond static genomic and transcriptomic measurements [29].

### 2.3 Protein‐Based Diagnostic Approaches

Among protein‐based methodologies, immunohistochemistry (IHC) remains the most widely used adjunctive diagnostic technique in dermatopathology for the evaluation of diagnostically challenging melanocytic lesions. IHC is a sensitive, rapid, and cost‐effective approach for detecting clinically relevant protein biomarkers [30, 31], including the BRAF p. V600E mutant protein [32], while preserving tissue morphology and histological context. Recent high‐plex spatial protein imaging of primary melanoma further highlighted this limitation by demonstrating substantial intratumoral variation in protein expression, suggesting that the reliability of individual markers may be strongly influenced by the spatial region sampled [33]. To overcome these limitations, mass spectrometry (MS)‐based proteomics enables the simultaneous, identification and quantification of thousands of proteins, providing a more comprehensive characterization of tumor biology. Importantly, mass spectrometry is not a novel technology in clinical diagnostics, as it is already routinely used in microbiology for pathogen identification (e.g., MALDI‐TOF MS) [34], and it is emerging as a promising complementary approach in liquid biopsy analyses as well [35].

Bulk proteomics measures proteins from homogenized tissue, offering deep and unbiased proteome coverage, typically identifying several thousand proteins per sample with a relatively low per‐sample cost compared to spatially resolved methods (Table 1) [27]. Several bulk proteomic studies have thus characterized primary melanoma at scale [29, 36, 37, 38, 39], but its application to diagnostically borderline or histologically ambiguous melanocytic lesions remains largely unaddressed. This reflects the practical difficulty of homogenizing small, morphologically heterogeneous lesions without diluting the tumor‐specific signals from adjacent reactive or benign tissue [40].

Its fundamental limitation for diagnostic purposes is that homogenization averages signal across all cell types and tissue compartments simultaneously, meaning that a small but biologically committed focus of early malignancy within a largely benign lesion may be diluted below the threshold of detection [41, 42]. This is a critical limitation for borderline melanocytic lesions, where the clinically relevant question is often not about the bulk composition of the biopsy, but about what is happening in a specific, morphologically atypical focus.

However, recent AI‐driven developments are beginning to overcome one of the fundamental limitations of bulk proteomics: The loss of spatial and cellular context. By integrating digital pathology, deep‐learning‐based tissue segmentation, cell‐type deconvolution, and proteomic reference signatures, bulk protein‐expression profiles can increasingly be computationally attributed to specific cellular and tumor‐microenvironment compartments. This creates an opportunity to transform conventional bulk proteomics into spatially informed molecular information, linking protein expression to tumor, stromal, immune, and other histologically defined compartments [43, 44, 45].

Single‐cell proteomics extends conventional bulk proteomics by enabling protein profiling at the resolution of individual cells. Unlike single‐cell transcriptomics, it directly measures proteins, the primary functional effectors of the cell, but currently remains limited by low protein input, reduced proteome depth, relatively low cellular throughput, and high technical complexity [27, 46]. Consequently, single‐cell proteomics is presently used primarily as a research tool rather than a routine diagnostic approach.

## 3 Spatial Proteomics in Borderline Melanocytic Lesions

Spatial proteomics as a protein‐based tool occupies a complementary position by preserving tissue architecture while directly measuring protein expression within histologically defined regions [41, 47]. It combines the spatial specificity needed for diagnostic work on heterogeneous lesions with the functional relevance of protein‐level data [47, 48].

### 3.1 Laser‐Captured Microdissection‐Based Spatial Proteomics and Its Current Application

One of the most widely used workflows for spatial proteomics combines laser capture microdissection (LCM) with mass spectrometry (MS), representing the most established approach for spatially resolved quantitative proteomic analysis of FFPE tissues [49, 50]. A typical experiment proceeds through five stages. FFPE sections (2–10 μm) are mounted on membrane‐coated (PEN) slides, deparaffinized, and stained (H&E or immunofluorescence) [27, 49]. Regions of interest are then annotated, increasingly via AI‐based digital pathology (e.g., QuPath deep‐learning segmentation), into tumor, stroma, and TIL compartments [5, 49]. Annotation accuracy directly determines the purity of the downstream proteomic signal, which is particularly important in borderline lesions where the morphological tumor and reactive distinction is itself uncertain. LCM platforms (ZeissPALM or Leica  LMD7) then physically isolates the annotated regions (typically ~500–1,000 cells per region for reliable protein detection), followed by trypsin digestion and LC–MS analysis. Modern DIA workflows (e.g., dia‐PASEF on timsTOF instruments) identify roughly 3,000–8,000 proteins per sample, with processing via DIA‐NN [49]. Finally, proteomic data are integrated with digital pathology features and clinical metadata using differential expression, pathway enrichment, and machine‐learning classification to derive diagnostic or prognostic signatures (Figure 2). These outputs require external validation before clinical claims can be made, a step most published studies have not yet completed.

**FIGURE 2:**
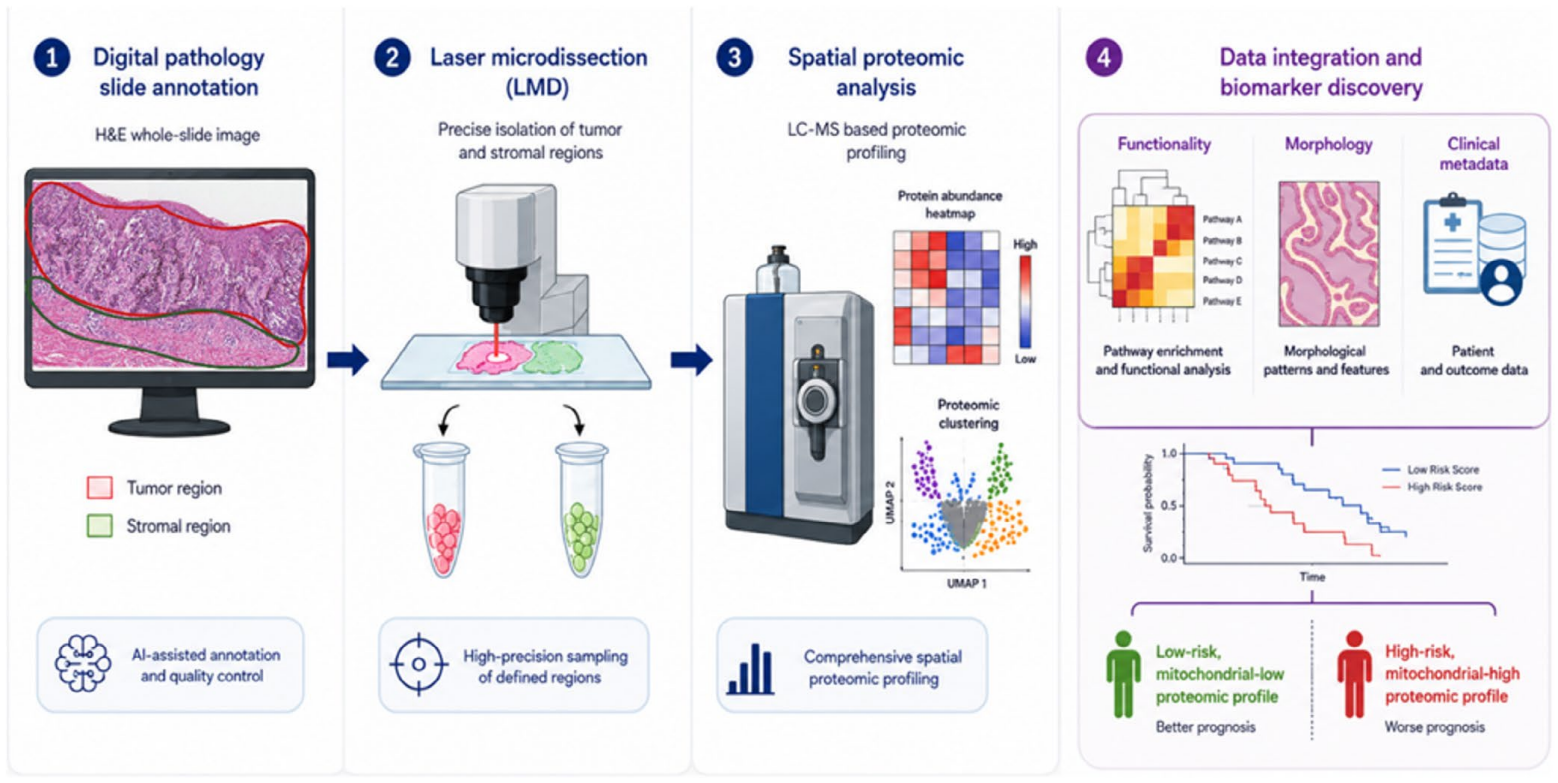
Spatial proteomics workflow for biomarker discovery in melanoma with laser‐captured microdissection. Whole‐slide H&E images are digitally annotated to define tumor and stromal compartments, which are subsequently isolated by laser microdissection (LMD). The dissected regions undergo LC–MS‐based spatial proteomic analysis to generate quantitative protein expression profiles. Integration of proteomic signatures with histopathological characteristics and clinical metadata enables the identification of candidate biomarkers and molecular risk profiles. The figure was created with BioRender [9].

The most clinically relevant question, whether proteomics can distinguish early melanoma from benign or borderline melanocytic lesions at the time of diagnosis. Importantly, LCM‐MS–based proteomic analysis of melanoma subtypes, nevus‐associated lesions, and nevi identified a small set of proteins capable of distinguishing melanoma from benign nevi [51], highlighting the diagnostic potential of LCM‐based proteomics for differentiating early melanoma from benign melanocytic lesions. Beyond diagnostic classification, the integration of AI‐based digital pathology, LCM, and quantitative proteomics has enabled spatially resolved molecular profiling of tumor and stromal compartments in early‐stage primary melanomas and other tumors (e.g., metastatic colon cancer) as well [41, 52]. This approach revealed distinct proteomic alterations associated with melanoma recurrence, including increased mitochondrial translation and oxidative phosphorylation and suppression of immune‐related processes, highlighting the potential of LCM‐based proteomics to identify molecular features associated with recurrence risk and support prognostic stratification of patients with early‐stage melanoma [49]. Moreover, in our previous studies, this multimodal strategy identified distinct biological signatures and spatially resolved molecular differences between tumor compartments in metastatic colon cancer [52]. For instance, proteomic analyses further revealed metabolic reprogramming, suppressed antigen presentation, and stage‐specific activation of inflammatory, angiogenic, epithelial–mesenchymal transition (EMT), and PI3K/AKT/mTOR–MYC signaling pathways, consistent with proliferative and therapy‐resistant colon cancer phenotypes. Collectively, these studies establish a biologically plausible proteomic basis for distinguishing early‐stage tumors from benign and borderline lesions, but they do not yet constitute a validated diagnostic framework.

### 3.2 Role of Imaging Mass Spectrometry in Distinguishing Borderline Lesions

Imaging mass spectrometry (IMS), most commonly performed using matrix‐assisted laser desorption/ionization imaging mass spectrometry (MALDI‐MSI), generates spatially resolved molecular maps directly from tissue sections without prior microdissection. Following matrix deposition, the laser systematically scans the tissue surface pixel by pixel, inducing desorption and ionization of analytes and generating a mass spectrum at each spatial coordinate. These spatially registered spectra are subsequently reconstructed into molecular ion maps. Currently, modern imaging mass spectrometry (IMS) platforms achieve spatial resolutions of approximately 10 μm under optimized conditions, enabling single‐cell and subcellular mapping of spatial molecular landscapes. This capability allows the simultaneous visualization of proteins, metabolites, lipids, and other molecular species directly within their native tissue architecture, providing unprecedented insight into cellular function and tumor biology. Its main trade‐offs are lower molecular specificity, and signatures are often defined as unidentified mass‐to‐charge peaks rather than named proteins [53]. However, in one of the largest imaging mass spectrometry (IMS) studies, a proteomic signature distinguished melanoma from benign nevus with > 98% accuracy in a training cohort (*n* = 113) and 93% accuracy in an independent validation set (*n* = 90), across 203 lesions total [54].

### 3.3 Deep Visual Proteomics and Its Clinical Role

Single‐cell and spatial resolution are not mutually exclusive. Deep Visual Proteomics (DVP) integrates AI‐guided image segmentation, automated single‐cell/single‐nucleus laser microdissection, and ultra‐sensitive mass spectrometry to achieve spatially anchored, single‐cell‐resolved proteomic profiling directly from FFPE archival tissue [50]. Rather than relying solely on manually defined region, DVP uses deep‐learning‐based image analysis to identify and isolate individual cells or nuclei of a given phenotype across a slide, then profiles their proteomes preserving their original spatial context. This represents the most direct technical convergence of AI‐based digital pathology segmentation and quantitative proteomics and has substantial potential for integration into the clinical workflow. Although, to our knowledge, DVP has not yet been applied specifically to the differential diagnosis of borderline melanocytic lesions, it is currently constrained by throughput and cost. This approach points toward a future workflow in which cell‐type‐resolved proteomic signatures could be generated for precisely the ambiguous single‐cell populations that make borderline melanocytic lesions diagnostically challenging.

### 3.4 Limitations

The infrastructure (LCM platforms, high‐resolution MS, computational capacity) of spatial proteomics remains confined to specialized research centers. Per‐sample costs are high relative to established molecular tests, though automation is progressively reducing both cost and variability. Turnaround times ranging from days to weeks remain incompatible with routine diagnostic timelines, while the risk of overinterpretation and batch effects represents additional barriers to clinical implementation [55]. Sample preparation of tumor tissue remains a labor‐intensive and time‐consuming procedure, representing a weak link in the overall analytical workflow. Spatial proteomics therefore currently performs best as a discovery platform rather than a clinically deployable diagnostic, but given the pace of technical development and the accuracy already demonstrated in melanoma vs. nevus discrimination, it has the potential to become a complementary layer alongside existing standards of care in borderline melanocytic lesions. The critical next step is the industrial scaling and full automation of the platform, enabling standardized, high‐throughput workflows that deliver reproducible spatial tumor profiling and clinically actionable reports at true single‐cell resolution. This capability is now emerging as a strategic priority for the next generation of spatial biology platforms being developed by leading technology manufacturers. We are currently advancing a technology development program in collaboration with TECAN, establishing an automated, end‐to‐end process workflow. The platform converts solid tumor tissue through a standardized multistep procedure into a liquid‐phase sample suitable for downstream protein expression analysis [56].

## 4 Implementation of Spatial Proteomics to Routine Clinic and Decision‐Making

### 4.1 The Role of Proteomic Plasticity in the Diagnosis of Borderline Lesions

Unlike DNA mutations, which are largely stable across time and tissue location, protein expression is continuously shaped by the local microenvironment, inflammatory state, metabolic activity, tissue handling conditions, and the evolving cellular composition of the lesion [57]. For example, the microenvironment of a melanoma is an always‐changing entity with tumor cells, cancer‐associated fibroblasts, endothelial cells, and immune cells. The cell and tissue composition of the sample can also lead to different proteomic profiles, since a “tumor” sample that contains 40% stroma will produce a very different proteomic profile than one containing 10% stroma, even if the tumor cells themselves are molecularly identical [57]. This is precisely why spatial proteomics, through region‐specific annotation and dissection, is conceptually better suited to this diagnostic task than bulk analysis. Moreover, the boundary between atypical melanocytes and reactive stromal or inflammatory changes is not always morphologically clear, particularly in borderline lesions. For instance, melanocytic lesions can be associated with variable degrees of inflammatory infiltration or solar elastosis [58, 59], which can produce substantial proteomic signal that may overlap with or mimic the immune and metabolic signature of malignancy.

Tissue handling itself is a further, technical source of proteomic variability distinct from biological plasticity. For example, the laser‐tissue interaction during LCM generates localized thermal effects at the cut margin that can degrade or denature proteins if laser power, pulse duration, and cutting parameters are not carefully calibrated to the tissue type and section thickness, potentially introducing artifactual signal loss at the tumor‐stroma interface most relevant to borderline lesions [60, 61]. Automation of upstream sample preparation steps, including deparaffinization and lysis, has been shown to reduce this variability by minimizing manual handling time and operator‐dependent inconsistency, thereby limiting both bias and protein loss across a cohort [56, 62].

### 4.2 Proteomic Signatures of Melanoma Progression to Potential Clinical Decision Support

A substantially larger body of proteomic evidence in melanoma derives from studies of disease progression rather than primary diagnosis [29, 36, 37, 38, 49]. One of the most consistently replicated findings across proteomic studies of melanoma progression is the enrichment of mitochondrial metabolic pathways, particularly oxidative phosphorylation (OXPHOS) and the tricarboxylic acid (TCA) cycle, in aggressive, recurrent, and metastatic tumors, a metabolic phenotype described in recent studies as “mitochondrial‐high” [49, 63]. These signatures are identified by quantitative MS‐based proteomics through differential protein expression and pathway enrichment analyses relative to appropriate reference groups [49, 63], rather than representing a single biomarker or binary molecular classification. It is important to be explicit that these signatures address a different clinical question from the one facing borderline lesions. They were derived by comparing recurrent or metastatic melanomas against non‐recurrent or primary tumors [49], all already diagnosed as melanoma, and therefore describe aggressive biological behavior within confirmed disease rather than distinguishing melanoma from a benign or intermediate melanocytic proliferation. Moreover, a BRAF V600E mutation can be present from the earliest stages of malignant transformation, but it does not predict which BRAF‐mutant tumor will remain indolent and which will progress rapidly [64]. The mitochondrial enrichment pattern associated with aggressive BRAF‐mutant melanoma, by contrast, can represent an active functional state, a metabolic adaptation to the tumor microenvironment, that may more directly reflect current biological behavior [63]. Although these spatially resolved proteomic signatures are not yet validated for clinical decision‐making and not for the differential diagnosis of borderline melanocytic lesions, their prospective evaluation could support future risk stratification and the development of complementary decision‐support tools for surveillance, treatment selection, and clinical‐trial prioritization (Table 2).

**TABLE 2:** Clinically grounded use cases for spatial proteomics in melanoma decision support.

Clinical question	Current standard	Spatial proteomic contribution	Potential decision impact	Readiness level
Borderline lesions	Dermoscopy, expert dermatopathology, IHC, selected molecular tests	Compartment‐specific tumor, stromal, and immune processes	Second‐opinion triage; prioritize ancillary testing	Early translational
Early‐stage recurrence risk	Breslow thickness, ulceration, mitotic rate, SLNB/AJCC staging	Mitochondrial translation, OXPHOS‐high and immune‐low tissue states	Surveillance intensity; SLNB/adjuvant therapy discussion; trial enrichment	Retrospective cohort signal
Metastatic progression	Imaging, staging, BRAF/NRAS/KIT testing, histopathology	Map metabolic rewiring and immune‐suppressed niches across lesions	Biopsy selection; identify aggressive biology; inform molecular review	Cohort‐level translational
Immunotherapy response	Clinical factors, LDH, stage, tumor burden, mutation status; limited routine biomarkers	Candidate protein panels and immune/metabolic state readouts	Support trial design and tumor board nuance, not standalone treatment selection	Hypothesis‐generating
Tissue‐to‐blood extension	Imaging and clinical follow‐up	Use tissue‐anchored signatures to guide future plasma or EV assays	Non‐invasive monitoring after prospective validation	Exploratory

Abbreviations: AJCC, American Joint Committee on Cancer; EV, extracellular vesicle; IHC, immunohistochemistry.

Unlike a genomic mutation, which is typically present or absent, proteomic abundance measures exist on a continuous scale, and early molecular data suggest that borderline lesions may occupy a spectrum between clearly benign and clearly malignant profiles rather than sorting into two discrete clusters. Translating such continuous or probabilistic output into a binary clinical action therefore requires prespecified risk thresholds, analogous to quantitative recurrence‐risk scores already used to stratify surveillance intensity, rather than relying on a single cutoff separating ‘benign’ from ‘malignant’.

For example, a case of a borderline lesion with a proteomic profile of consistent early malignant transformation, specifically showing emerging mitochondrial enrichment and early immune microenvironment remodeling, might justify wider excision margins, mandatory nodal staging, more intense surveillance, and even, in the absence of unequivocal histological malignancy, reducing the probability of local recurrence from a biologically committed lesion that was morphologically underclassified. However, this remains entirely speculative at present. No existing proteomic classifier has been prospectively validated against long‐term clinical outcomes, and acting on molecular signals of uncertain significance risks unnecessary intervention and molecular overdiagnosis.

In a similarly exploratory sense, for patients with early‐stage melanoma at high molecular risk of recurrence despite favorable conventional staging, proteomic risk stratification could identify candidates for adjuvant therapy trials who would not currently meet enrollment criteria based on AJCC stage alone (Figure 2).

Another consideration regarding clinical practice is whether spatial proteomics might increase diagnostic complexity or generate a new form of molecular overdiagnosis. Because proteomic profiling can detect subtle, low‐magnitude molecular deviations [41] that may fall short of unequivocal malignancy. There is a risk that borderline or indeterminate molecular findings could prompt escalated management (wider excision, nodal staging, intensified surveillance) in lesions that would otherwise have remained clinically indolent. On the other hand, mitigating this risk will require that any future proteomic classifier be validated against long‐term clinical outcomes, not only against a binary benign/malignant histological reference standard. Decision thresholds should be explicitly calibrated to avoid triggering intervention on molecular signals of uncertain clinical significance. From our experience, this objective can be achieved through the establishment of large, well‐annotated tumor databases that integrate multimodal molecular and clinical data, enabling the development of AI‐driven predictive models for precision diagnostics, prognostication, and therapeutic decision support.

### 4.3 Future Directions and Conclusions

Realizing the clinical potential of spatial proteomics in borderline melanocytic lesions will require progress on several fronts. Continued automation of sample preparation and DIA acquisition should narrow the current gap in turnaround time and cost, while advances in single‐cell and AI‐based deep visual proteomics workflows are expected to increase throughput and reduce the input material required per region. Methodologically, the field must move beyond cohorts of unambiguous melanoma and nevi toward prospectively collected series enriched for diagnostically indeterminate categories, where clinical need is the greatest. Clinically, spatial proteomics is likely to add the most value not as a standalone assay but as a complementary layer within a multimodal framework. It may not be far off that multidisciplinary tumor board decision‐making for borderline melanocytic lesions is routinely guided by the integration of spatial proteomic, artificial intelligence–derived digital pathology, and genetic information into a single decision‐support output, rather than by any one modality in isolation. The immediate goal, however, is not to position proteomics as a diagnostic replacement, but to define where functional tissue‐state information, generated within standardized, reproducible workflows, can strengthen clinical interpretation beyond morphology alone.

## Funding

This work was supported by grants from the Berta Kamprad Foundation, Lund, Sweden (FBKS‐2025‐18 [671]). This work was conducted under the auspices of a Memorandum of Understanding between the European Cancer Moonshot Center in Lund and the U.S. National Cancer Institute's International Cancer Proteogenome Consortium (ICPC), which promotes international collaboration and the open sharing of proteogenomic cancer datasets. The study was also carried out in collaboration with the U.S. National Cancer Institute's Clinical Proteomic Tumor Analysis Consortium (CPTAC).

## Disclosure

During the preparation of this manuscript, the authors used ChatGPT (OpenAI, GPT‐5.1 [65]) and Claude.ai (Anthropic, Claude [66]) solely to assist with language editing and to improve the clarity and readability of the text. All AI‐assisted content was critically reviewed, revised, and approved by the authors, who take full responsibility for the accuracy and integrity of the final manuscript.

## Conflicts of Interest

The authors declare no conflicts of interest.

## Data Availability

The data that support the findings of this study are available from the corresponding author upon reasonable request.
